# Endovascular Treatment of the Ascending Aorta: is this the Last Frontier in Aortic Surgery?

**DOI:** 10.21470/1678-9741-2019-0317

**Published:** 2019

**Authors:** Eduardo Keller Saadi, Ana Paula Tagliari, Rui M. S. Almeida

**Affiliations:** 1 Departamento de Cirurgia Cardiovascular, Faculdade de Medicina, Universidade Federal do Rio Grande do Sul, Porto Alegre, RS, Brazil.; 2 Departamento de Cirurgia Cardiovascular, Hospital São Lucas, Pontifícia Universidade Católica do Rio Grande do Sul, Porto Alegre, RS, Brazil.; 3 Departamento de Cirurgia Cardiovascular, Faculdade de Medicina, Centro Universitário Fundação Assis Gurgacz, Universidade Estadual do Oeste, Cascavel, PR, Brazil.

**Keywords:** Ascending Aorta, Cardiac Surgery, Endovascular Procedures, Endovascular Surgery, Thoracic Endovascular Repair

## Abstract

Regardless the successful treatment of the descending aorta with endovascular prosthesis, for the ascending aorta segment, because of several anatomic and physiologic issues, this technique has been considered an alternative only for high-risk or inoperable patients. Despite restricted indications, hundreds of treatments have been performed worldwide, demonstrating its safety and reproducibility if it is done in high-quality centers. Therefore, understanding patients’ selection criteria and technique limitations are critical to its application.

**Table:** 

Abbreviations, acronyms & symbols	
BCT	= Brachiocephalic trunk
CT	= Computed tomography
ESC	= European Society of Cardiology
GREAT	= Global Registry for Endovascular Aortic Treatment
SG	= Stent graft
STJ	= Sinotubular junction
TAAD	= Type A aortic dissection
TEVAR	= Thoracic aorta endovascular repair

## INTRODUCTION

The concept of treating the ascending thoracic aorta with endoprosthesis emerged after the first-in-man successful case described by Dorros et al.^[1]^ in 2000. Since then, several studies have reported the safety and effectiveness of the ascending aorta endovascular repair.

Despite this, contrasting with the descending aorta, where thoracic aorta endovascular repair (TEVAR) is well established^[2,3]^, for proximal aortic disease, open surgery is considered the gold standard for treatment, although less invasive procedures are achieving an important role, especially in high-risk or inoperable patients.

**Fig. 1:**
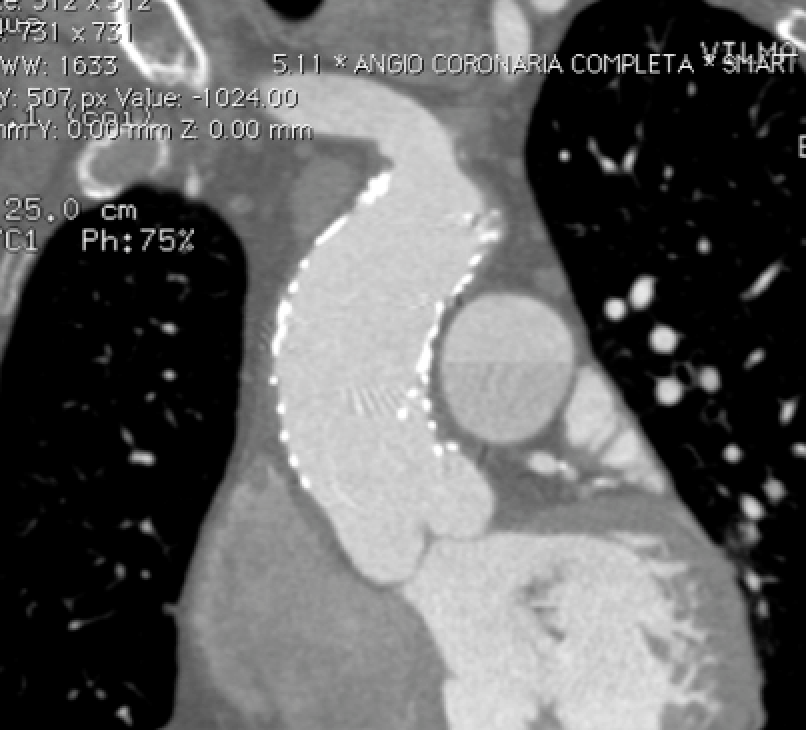
Computed tomography post stent graft in ascending aorta.

**Fig. 2:**
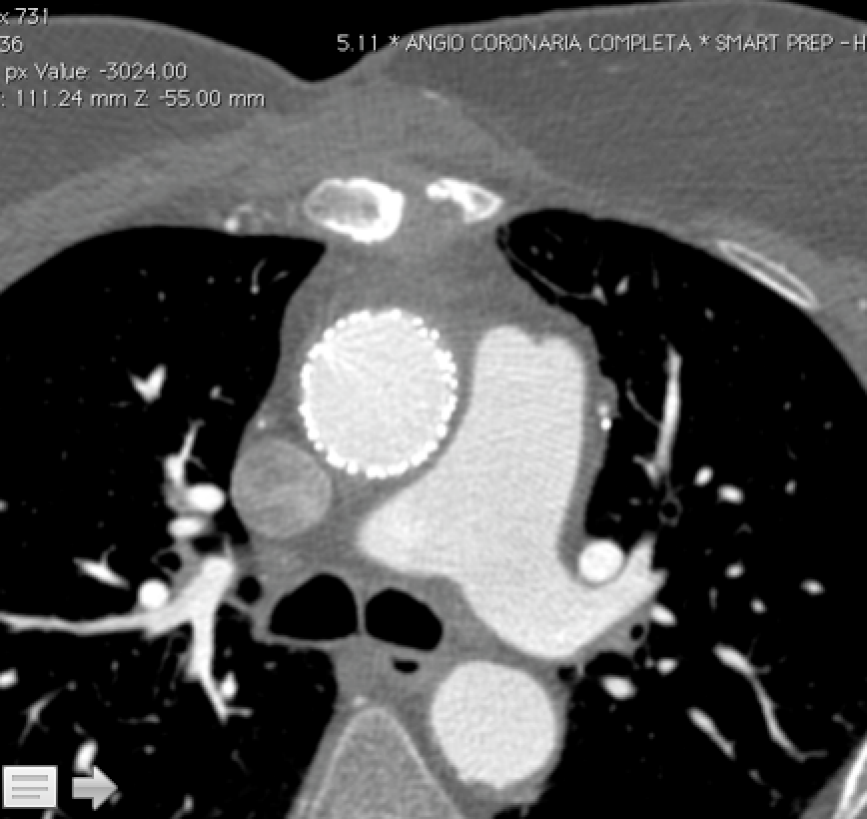
Computed tomography, axial view, post stent graft in ascending aorta.

The need for an alternative treatment becomes even more relevant when considering that more than 20% of the patients with type A aortic dissection (TAAD) are deemed inoperable^[4]^. In this context, ascending TEVAR could have potential advantages, such as absence of thoracotomy incision and need for partial or total extracorporeal circulatory support, as well as lower hospital morbidity rates and shorter length of hospital stay when compared to open surgery^[5,6]^. This was already well established on our initial experience^[7]^ (Figures 1 and 2).

### Indications and Planning

According to the 2014 European Society of Cardiology (ESC) Guidelines on diagnosis and treatment of aortic diseases, the use of TEVAR should be decided on an individual basis, according to the anatomy, pathology, comorbidity, and anticipated durability criteria, using a multidisciplinary approach (Class I, Level C)^[8]^.

Planning the procedure, this guideline reinforces that it is mandatory to perform preoperative imaging and engineering analysis, with contrast-enhanced computed tomography (CT) representing the imaging modality of choice^[8]^.

**Fig. 3:**
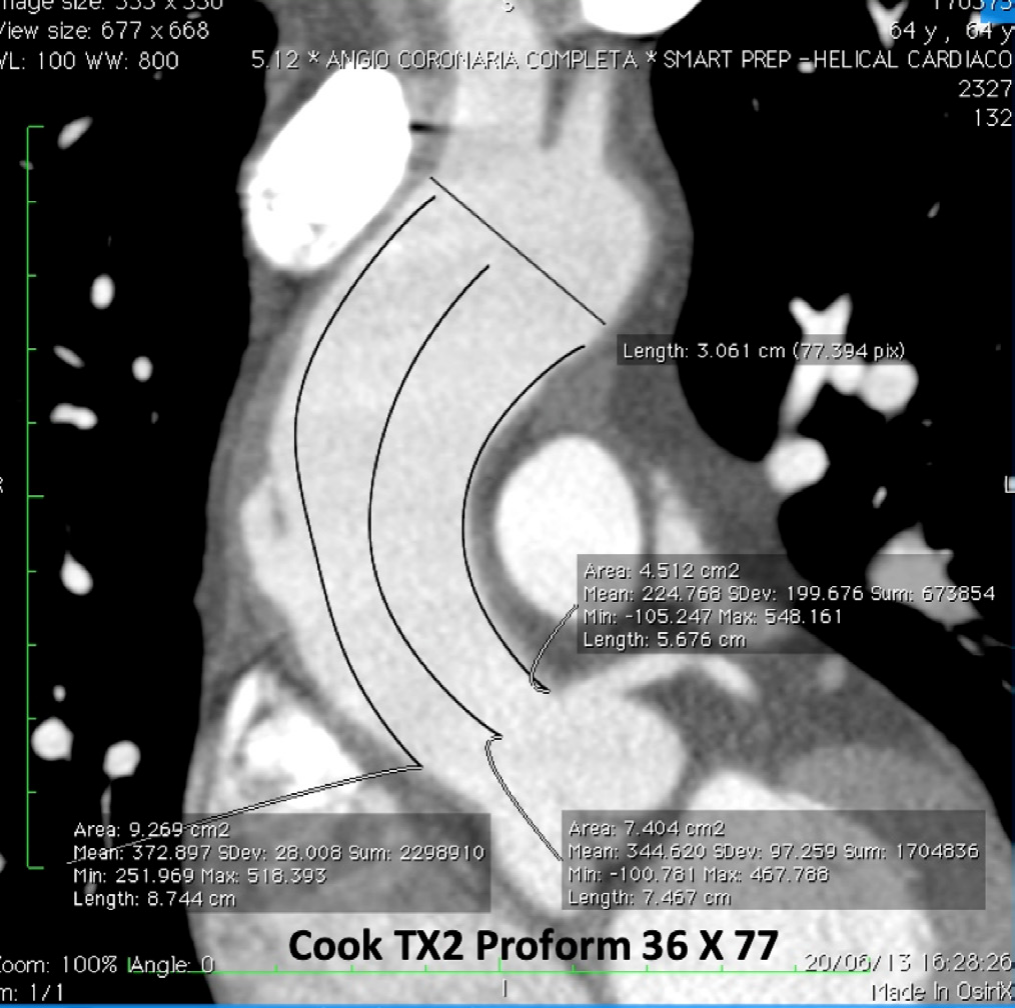
Ascending aortic dissection. Planning endovascular treatment with preoperative contrast-enhanced computed tomography.

CT planning is indispensable because the ascending aorta has some particular characteristics different from the other segments, like its size, shape, hemodynamic forces, and proximity to other anatomic structures (Figure 3). Moreover, more complex anatomy and pathologies are frequently detected in the ascending segment^[9]^.

### Surgical Technique

As ascending aorta endovascular treatment involves several challenges and potential catastrophic complications^[10]^, these procedures should be done in a hybrid room, with cardiopulmonary bypass available, as well as transesophageal echocardiogram and transvenous pacemaker, and by a surgical team well trained on both procedures (conventional and endovascular)^[11]^.

Regarding anatomical suitability criteria, a landing zone of at least 2 cm, an aortic diameter < 40 mm^[8-11]^, an absence of grade 3/4 regurgitation, and iliofemoral vessels with a diameter > 7 mm and an angulation < 90° (for 24-French delivery system) have been required. Besides this, in cases of TAAD, a distance from the entry tear to the coronary ostia > 20 mm is also mandatory^[12-14]^.

As mentioned by Muetterties et al.^[15]^, these criteria are critical because the proximity between the endoprosthesis proximal landing zone and the aortic valve or the coronary ostia could result in aortic insufficiency or myocardial infarction if the stent graft (SG) is accidentally deployed in a proximal position. Likewise, distal stent migration could lead to brachiocephalic artery occlusion, resulting in stroke. Therefore, the ideal candidates for an ascending TEVAR procedure are patients with focal aortic defects, including pseudoaneurysms and ascending aortic dissections with focal entry tears in the middle third of the ascending aorta.

Conformability of the device to fit the aorta curvature and to provide a better seal along entry tears is also an important characteristic. Thus, for chronic disease, it is recommended an oversizing by about 20%, instead of the typical 10% recommended for acute dissections^[16]^.

According to Moon et al.^[17]^, for patients with TAAD evaluated by CT, other endograft suitability criteria are an adequate proximal landing zone (sinotubular junction [STJ] ≤ 38 mm), fenestration distal to STJ, minimum distance between intimal fenestration and STJ ≥ 10 mm, and absence of coronary bypass grafts originating from the ascending aorta.

Moreover, based on findings of the Global Registry for Endovascular Aortic Treatment (GREAT), Piffareti et al.^[18]^ demonstrated that the most adequate SG size was selected using a three-dimensional centerline reconstruction workstation. For aortic dissection and pseudoaneurysm, an SG diameter equal to the wall-to-wall diameter without oversizing was used. For aortic aneurysms, the SG was oversized 20% based on the larger of two measurements performed at the STJ or at the distal ascending aorta. The authors also described that the SG should be ideally deployed under temporary overdrive cardiac pacing, generally, 190 beats/min.

The most common access sites are the transfemoral, transapical, or axillary arteries, being the last two a potential alternative when the anatomy is unfit for a standard retrograde approach^[19]^.

### Available Devices

**Fig. 4:**
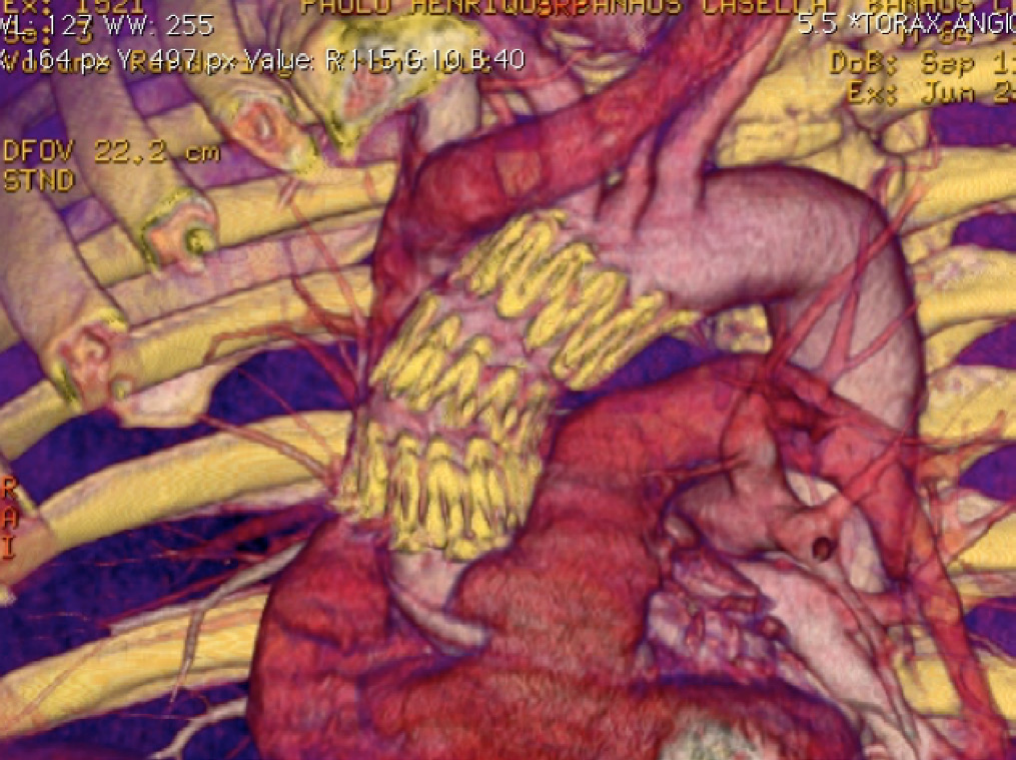
Three-dimensional computed tomography post stent graft in ascending aorta.

**Fig. 5:**
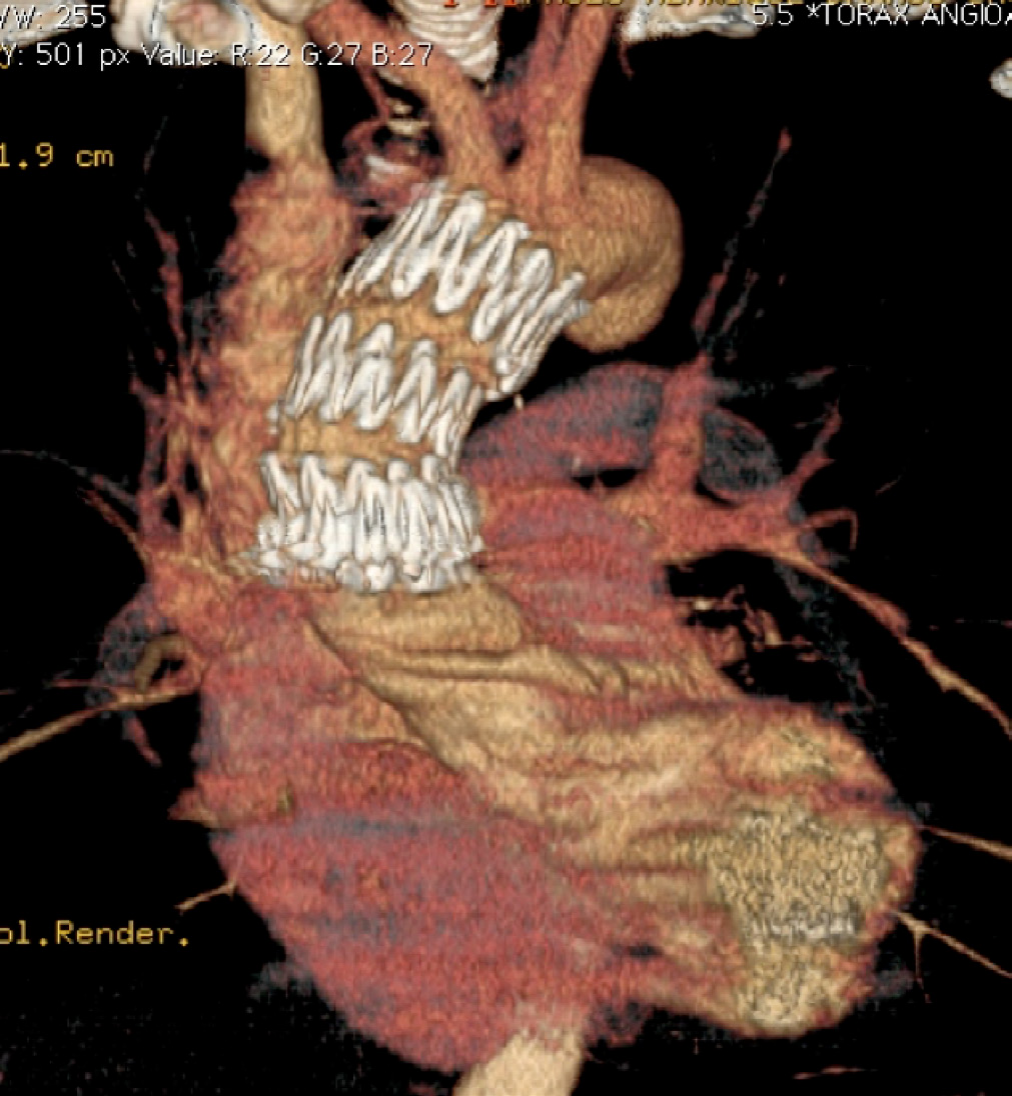
Three-dimensional computed tomography post stent graft in type A aortic dissection.

TEVAR in the ascending aorta segment can be performed with dedicated ascending aortic SG (Zenith Ascend TAA Endovascular Graft, William Cook Europe, Bjaeverskov, Denmark), a standardized device or a surgeon-modified thoracic SG^[20]^ (Figures 4 and 5).

Limitations to use standard thoracic SG in TAAD are the location of the intimal tear, presence of higher-grade aortic valve insufficiency, and an aortic diameter > 38 mm^[21]^.

A concerning point is that the majority of the SGs currently available on the market are too long for ascending aorta deployment or have a too long nose cone, which could interfere with the native aortic valve during the deployment, leading to a necessity of customization or intraoperative modification of the devices^[17]^ (Figure 4). Some devices also have a delivery system length that does not reach ascending aorta in big patients or patients with angulated aortas.

### Procedure Complications

The most important complications in ascending TEVAR are cardiac tamponade, stroke, endoleak, stent distal migration, residual dissection, aneurysm, left ventricle perforation, injury and dissection of the aortic root, and occlusion of the coronary arteries^[22]^.

Some of the advantages in this procedure concern the fewer cases of endoleak, as stated by Khoynezhad et al.^[22]^, who highlighted that the absence of side branches in the ascending aorta makes type II endoleak not possible. The same applies to type III endoleak, in cases where just one graft is deployed, this complication is inexistent. Therefore, endoleaks observed in ascending aorta TEVAR are mainly type Ia and Ib.

### Available Outcomes

In terms of clinical results, in 2015, Roselli EE et al.^[9]^ published their own experience with 22 patients enrolled from 2006 to 2014 at the Cleveland Clinic. Cases of acute TAAD, intramural hematoma, pseudoaneurysm, chronic dissection, or aorta-cardiac fistula have undergone supracoronary ascending TEVAR procedure. Acceptable hospital mortality (13.6%) and an actual survival rate at 30 days, one year, and five years of 86%, 80%, and 75%, respectively, were demonstrated.

The same authors, in 2018, published a new article proposing a modification in the landing zone classification based on the evaluation of 39 patients, 36 of them managed with TEVAR. CT imaging analysis was performed, and the extent of aortic pathology was designated by the segmental proximity to the left ventricle, by diving the zone zero into three segments: a) zone 0A constitutes the aortic root and is defined proximally by the left ventricular outflow tract and distally by the distal edge of the coronary ostia; b) zone 0B constitutes the proximal half of the ascending aorta and is defined as the region between the distal coronary ostia and the right main pulmonary artery; c) zone 0C is the distal half of the ascending aorta extending from the right main pulmonary artery and including the brachiocephalic trunk (BCT)^[23]^.

Multivariable analysis of this cohort demonstrated a significantly higher hazard of mortality in diseases involving zone 0A *vs*. 0C (*P*=0.020) and in the elderly (*P*=0.026). At 30 days, one year, and five years, the overall survival rate was 81%, 74%, and 64% and the freedom from reintervention was 85%, 77%, and 68%, respectively^[23]^.

Tsilimparis N et al.^[24]^ similarly reported their experiences with 24 high-risk unsuitable for open repair patients. Pathologies included acute aortic dissection, chronic dissection, and pseudoaneurysm. The 30-day survival rate was 79% and major adverse events occurred in six (25%) patients, with a reintervention rate of 12.5%.

Unifying the case and series reports, Muetterties et al.^[15]^ recently published a systematic review comprising 46 publications with a total of 118 patients treated for different aortic diseases: TAAD (50%), aortic pseudoaneurysms (30%), aortic aneurysm (5%), penetrating atherosclerotic ulcer (4.2%), or acute aortic rupture (2.5%). In this review, 13 different aortic SG of various designs were used, most commonly the thoracic stents (71%), abdominal cuffs (1%), and custom-made grafts (10%). In this analysis, femoral arterial access was used in 62.7%, transapical in 14.4%, carotid in 12.7%, and axillary in 6.8% of the patients. In an average follow-up of 17.2 months, type I endoleak was diagnosed in 18.6% (with 9.3% requiring reintervention), the rate of all-cause mortality was 15.2%, aorta-related mortality was 5%, conversion to open surgery occurred in 3.4%, and cerebrovascular complications occurred in 3.4% of the patients^[15]^.

Another similar meta-analysis of 30 articles identified a high rate of prior cardiac operation (67%) in a total population of 119 patients managed with TEVAR in the ascending aorta. Technical success was achieved in 95.5%, conversion to open repair in 0.7%, neurologic events in 1.8%, myocardial infarction in 0.8%, and 30-days mortality was 2.9%^[15]^.

Analyzing exclusively non-dissected ascending aorta disease, systematic review encompassing 67 patients (mean age 65±17 years) reported early mortality of 2.9%, endoleak rate of 13.4%, stroke of 4.8%, and non-ST-elevation myocardial infarction of 1.5%. Six patients (8.9%) required reintervention after endovascular repair (five required open surgery and one endovascular reintervention for an endoleak). The authors observed that the devices used were off-the-shelf, custom-designed, or modified-design SG and the average length of the SG was 66.9±25.9 mm^[25]^.

Even in cases where the aortic valve is compromised, the concept of a proximal transcatheter aortic valve connected to an uncovered portion of an SG (“endo Bentall”) is emerging. This could result in shorter landing zones and, therefore, could increase the number of patients suitable for endovascular treatment^[26]^.

**Fig. 6:**
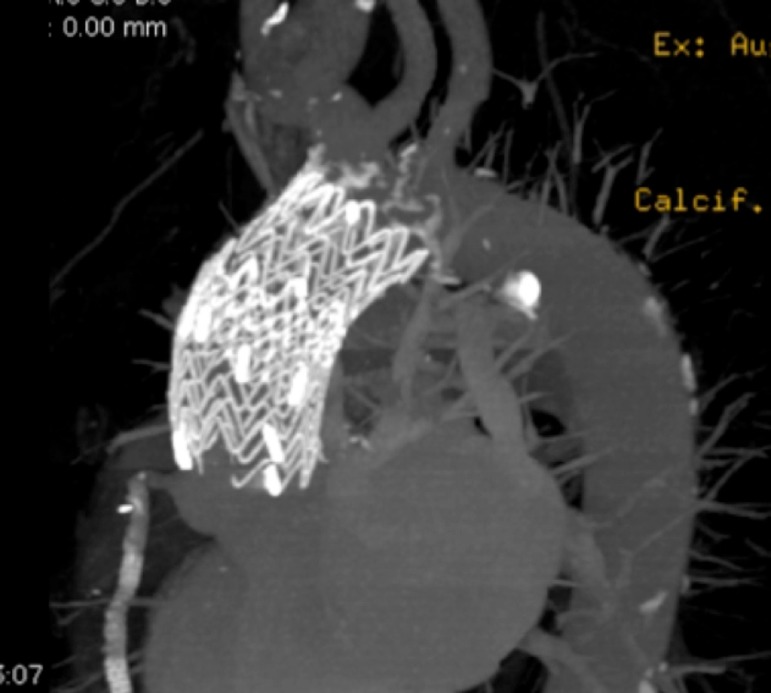
Use of two stent grafts for correction of ascending aorta pseudoaneurysm and coronary stent on right coronary artery.

In terms of national experiences, in 2011, Saadi et al.^[27]^ reported two successful lifesaving cases of patients with ascending aorta pseudoaneurysm managed with implantation of SG together with percutaneous coronary intervention secondary to ischemic disease, proving the feasibility of the combined method^[27]^ (Figure 6).

## CONCLUSION

Ascending aorta TEVAR represents the final frontier of endovascular therapy and it is well proven to be a lifesaving procedure in selected patients, using customized or off-the-shelf endoprosthesis. There are still several anatomical, physiological, and technical challenges, such as the short distance between coronary arteries and BCT, aortic valve regurgitation and obstruction, high hemodynamic forces, angulation, coronary arteries, and cerebral perfusion. Potential serious complications like aortic dissection, rupture, coronary and BCT obstruction, and aortic valve damage require a well-trained multidisciplinary team in a hybrid room environment. Therefore, understanding patients’ selection criteria, limitations of the technology, and development of dedicated devices are critical to its application. Trials are needed to provide evidence-based data.

**Table:** 

Authors' roles & responsibilities	
EKS	Substantial contributions to the conception or design of the work; or the acquisition, analysis, or interpretation of data for the work; final approval of the version to be published
APT	Substantial contributions to the conception or design of the work; or the acquisition, analysis, or interpretation of data for the work; final approval of the version to be published
RMSA	Substantial contributions to the conception or design of the work; or the acquisition, analysis, or interpretation of data for the work; final approval of the version to be published
